# The influence of social capital on farmers’ green control technology adoption behavior

**DOI:** 10.3389/fpsyg.2022.1001442

**Published:** 2022-10-10

**Authors:** Zhong Ren, Zitian Fu, Kaiyang Zhong

**Affiliations:** ^1^Business School, Shandong Normal University, Jinan, China; ^2^School of Economics, Sichuan Agricultural University, Chengdu, China; ^3^School of Economic Information Engineering, Southwestern University of Finance and Economics, Chengdu, China

**Keywords:** social capital, green control technology, Probit model, endogenous, instrumental variable method

## Abstract

Relying on social capital to promote farmers’ adoption of green control technology is of great significance for the governance of rural environment and the realization of sustainable agricultural development. Based on the survey data of 754 farmers in Shandong Province, this paper uses the Probit model and the instrumental variable method to empirically analyze the impact of social capital on farmers’ green control technology adoption behavior. The results show that: social capital has a promoting influence on farmers’ green control technology adoption behavior; the influence of the three dimensions of social capital on farmers’ green control technology adoption behavior is in turn social norms, social networks, and social trust; social networks play an enhanced moderating role in the process of social trust and social norms promoting farmers’ green control technology adoption behavior; education level, the number of family labor force and annual family income level have a significant positive impact on farmers’ green control technology adoption behavior, while age has a significant negative impact. Therefore, the government should make full use of social capital to promote farmers to adopt green control technology.

## Introduction

For a long time, the high yield and income of Chinese agriculture have been dependent on the application of a good number of chemical inputs. The long-term excessive and inefficient application of chemical inputs pose a huge threat to the quality of agricultural products, rural ecological environment, and human life and health (Wang et al., 2022). In order to solve the negative externalities of chemical inputs application, the Chinese government has actively promoted green control technology (GCT) since 2006. GCT is an environmentally friendly technology widely used in European and American countries, and it is the sinicization of the concept of Integrated Pest Management (IPM) (Gao et al., 2017). GCT emphasizes the comprehensive utilization of ecological regulation, biological control, physical, and chemical inducement, and scientific drug use or their combination to effectively control pests and diseases, ensure ecological environment safety, promote agricultural cost savings and increase income, and minimize farmers’ dependence on chemical inputs (Yu et al., 2020; Niu et al., 2022). By the end of 2019, the coverage rate of GCT in China was only 37% (Ministry of Justice of the People’s Republic of China, 2020), and the application level was not high. How to effectively implement the promotion and application of GCT has become a key issue that needs to be solved urgently in the process of ensuring the quality of agricultural products and promoting the sustainable development of agriculture in China.

Many studies believe that farmers, as direct users and stakeholders of GCT, generally lack enthusiasm for adopting GCT, which makes it difficult to effectively break through the scale of promotion (Lou et al., 2021; Wu and Zhou, 2021). What factors prevent Chinese farmers from adopting GCT? From the perspective of the market, farmers are “rational economic people,” and the economic incentive mechanism is the main driving force for the promotion of agricultural technology (Griliches, 1957). Farmers’ adoption of GCT requires higher economic costs and certain risks. Farmers will adopt GCT only if the net benefit of GCT is greater than the net benefit of traditional chemical inputs (Martey et al., 2020; Yang et al., 2020). But at this stage, China’s green agricultural product market is not perfect, not only the market principle of “high quality and high price” for agricultural products is not reflected, but also the “lemon market” effect is caused by information asymmetry. The “invisible hand” cannot form an effective incentive (Nie et al., 2020). From the perspective of technology extension, training and technical support for farmers is an effective method to promote farmers to adopt GCT (Khanal et al., 2020; Liu et al., 2022). However, China’s government-led extension model is faced with many problems, such as limited extension resources, low quality of extension personnel, mismatch between extension content and farmers’ needs, and the extension effect is not ideal (Gao et al., 2020; Huang et al., 2022). From the perspective of the policy, although the government has issued a number of guiding documents such as “Opinions on Promoting Green Control of Crop Pests and Diseases” and “Key Points of Green Development of Agriculture and Rural Areas,” there is a certain gap between the actual effect and policy expectations due to the dispersion and uncertainty of rural environmental problems, as well as the existence of heterogeneity in the historical and cultural foundation of each village and the level of social and economic development (Li et al., 2019; Huang et al., 2021).

According to the theory of new economic sociology, individuals are embedded in the social structure, and their actions are bound to be restricted by sociological “embedded factors” (Granovetter, 1985). Rural China is a “relational” society based on blood, kinship and geography. Farmers’ technology adoption behavior is not only influenced by external factors such as policy and market, but also by the social capital embedded in the rural social environment (Gao et al., 2019). Social capital can promote farmers to break through the existing resource constraints and change the boundary between the adoption and not adoption of agricultural technology through mechanisms such as information dissemination, risk sharing, demonstration, and guidance (Castillo et al., 2021). In addition, the positive externalities of GCT can easily lead to collective action getting into trouble. Social capital can combine individual action with collective action, promote cooperation among individuals, reduce uncertainty in the environment of individual behavior choices, and effectively restrain individual “free ride” behavior (Wulandhari et al., 2022). Previous studies have confirmed the role of social capital in farmers’ domestic sewage treatment, overgrazing, irrigation technology (Hunecke et al., 2017; Zhang et al., 2020; Ding et al., 2021), but few people have investigated the impact of social capital on farmers’ adoption behavior of GCT. Will the society capital influence farmers’ GCT adoption behavior? If this kind of influence exists, what is its mechanism and direction? The answers to the above questions are of great practical significance for understanding the internal logic of farmers’ GCT adoption behavior, and formulating and perfecting the GCT promotion policy.

Based on the abundant research achievements of previous scholars, this paper tries to make the following explorations: firstly, most of the literatures focus on the influence of individual characteristics such as gender and education level (Rezaei et al., 2019; Steiro et al., 2020), subjective cognitive characteristics such as cognitive closure and environmental cognition (Abadi, 2018; Rezaei et al., 2020), and institutional policy characteristics such as technology promotion models and land ownership on farmers’ GCT adoption behavior (Gao et al., 2017; Wang et al., 2018, 2020), but less on the influence of social capital. In fact, besides the characteristics of general agricultural technology, GCT also has typical positive externalities, which can easily lead to “collective action dilemma.” The social capital hidden in the peasant group can effectively solve this dilemma through information transmission, demonstration effect, internal supervision and reciprocal cooperation. Therefore, this study examines the influence of social capital on farmers’ GCT adoption behavior from different dimensions, which broadens the research perspective. Secondly, the literature does not pay attention to the relationship between different dimensions of social capital. For example, Zhang et al. (2020) verified the influence of social trust, networks and norms on farmers’ domestic sewage treatment, but ignored the interaction between them. Based on the comparative analysis of the differences in the influence of social trust, social norms, and social networks on farmers’ GCT adoption behavior. This study further examines the moderating role of social networks in the influence of social trust and social norms on farmers’ GCT adoption behavior, which improves the depth of research content. Thirdly, the literature is not aware of the possible endogenous problems. For example, Ogunleye et al. (2021) ignored the endogeneity of social networks when analyzing the impact of social capital networks on farmers’ adoption of climate change adaptation strategies. This study eliminates the endogenous influence of social capital and farmers’ GCT adoption behavior on the regression results through instrumental variable method, which improves the accuracy of the research results.

The structure of the full text is as follows: Section “Introduction” introduces the implementation background of GCT in China. Section “Theoretical analysis and research hypothesis” introduces the theoretical framework of the influence of social capital on farmers’ GCT adoption behavior. Section “Research design” introduces the definition of variables, research methods, and data sources. Section “Estimation results and discussion” introduces empirical analysis and discussion of the results of the impact of social capital on farmers’ GCT adoption behavior. Section “Conclusion and policy suggestions” summarizes the main conclusion and policy implications.

## Theoretical analysis and research hypothesis

The concept of social capital originated in the field of sociology, which was first put forward and systematically expounded by Bourdieu (1986). He believed that social capital is a network of relationships that helps actors obtain actual or potential resources. Subsequently, Coleman (1988) defined social capital as a personal capital property characterized by social structural resources from a functional perspective. Putnam and Leonardi (1994) promoted social capital from the individual level to the collective level, and believed that social capital is a certain feature of social organizations, including social trust, social networks, and social norms. Since then, the academic research on the definition of social capital has gradually moved closer to social trust, social networks, and social norms. Therefore, the social capital in this paper refers to the mutual trust, relationship network and common values among farmers formed in rural long-term life contacts, which can be summarized into three dimensions: social trust, social networks, and social norms. Its influence on farmers’ GCT adoption behavior is as follows:

### Social trust and farmers’ green control technology adoption behavior

Social trust refers to the subjective probability that a social individual evaluates that other individuals will take a specific action in the future, and this evaluation will have an impact on the social individual’s own actions. To a certain extent, social trust determines whether farmers are willing to pay credit or rely on others’ suggestions to act, which will constrain or motivate farmers’ “free ride” psychology in collective action, and then encourage or inhibit farmers’ participation in collective action (Ma et al., 2022). Social trust can be further divided into interpersonal trust and institutional trust. Interpersonal trust, which uses the emotion between people as a bond, often occurs between relatives and friends, and has the characteristics of closeness and distance, which also causes the difference of trust strength. Good interpersonal trust can enhance farmers’ mutual identity, increase farmers’ willingness to share information resources, promote the flow and transformation of information, and reduce information asymmetry in farmers’ adoption of GCT (Granovetter, 1985). Institutional trust often depends on institutional environments such as legal, political and so on. In rural areas, Farmers’ institutional trust can be measured by their trust in village cadres (He et al., 2018). A higher level of institutional trust is conducive to enhancing the guarantee role of the government and village cadres, overcoming the farmers’ psychology of “uncertainty in adopting GCT,” restraining the generation of farmers’ opportunistic behavior and avoiding the “prisoner’s dilemma” (Cao et al., 2020). Based on this, this paper proposes the hypothesis:

H_1_: Social trust will promote farmers’ GCT adoption behavior.

### Social networks and farmers’ green control technology adoption behavior

Social networks are relatively stable social system formed by the interaction between individual members of society, which emphasizes the interaction and connection between people. According to the view of embeddedness, the individual’s behavior decision is not completely independent, but will be influenced by other members in the network (Granovetter, 1985). In China’s rural areas, such a social environment with complex local relations, the influence of farmers’ social networks on their behavior decisions is more obvious (Gao et al., 2022). Due to the heterogeneity of social networks, social networks can be further divided into strong ties and weak ties (Ostrom, 2010). Strong ties refer to the strong homogeneity of personal social networks and the close relationship between people, which put more emphasis on the strength of social networks. Due to the reciprocal motives among members, strong ties can reduce the cost of obtaining and analyzing information for farmers, and provide opportunities for mutual learning, exchange and help (Zheng and Luo, 2022). Weak ties refer to individuals with strong heterogeneity in their social networks and not close relationship between people, which can better reflect the breadth of social networks. Due to its relatively open nature, weak ties are more conducive to farmers to obtain more external information across strata, broaden farmers’ horizons, reduce cognitive bias, and promote collective action (Granovetter, 1973). Based on this, this paper proposes the hypothesis:

H_2_: Social networks will promote farmers’ GCT adoption behavior.

### Social norms and farmers’ green control technology adoption behavior

Social norms are the rules and principles that members of a group abide by without legal constraints. Individuals usually want to be recognized by the group and try to escape potential social criticism or sanctions from others (Abrahamse and Steg, 2013). Social norms can exert tangible or intangible pressure on members, and promote the behavior of group members to be consistent with the group (Heinicke et al., 2022). Social norms can be further divided into imperative norms and descriptive norms. Imperative norms refer to the behavioral standards that most people approve of and think should be taken or most people oppose and think should not be take. Imperative norms motivate individuals to choose behaviors consistent with the behavior of the majority through social constraints or rewards (Li et al., 2021). Descriptive norms refer to the behavior standards formed by the behaviors that most people have taken or are taking in specific situations. When individuals lack sufficient information to make judgments, they will refer to the behavior of other social members as the basis for their own behavior, and show behaviors similar to other social members, showing an obvious “herd effect” (Asch, 1956). Based on this, this paper proposes the hypothesis:

H_3_: Social norms will promote farmers’ GCT adoption behavior.

### The moderating role of social networks

Social trust is the product of universal contact and communication between individuals, which is undoubtedly embedded in social networks and deeply restricted and influenced by social networks (Huang et al., 2021). There are two mechanisms by which social networks affect social trust: the first is the “resource” mechanism based on the “social resource theory.” The degree of individual trust in others depends on the ability to bear losses. Farmers usually obtain social resources from social networks. Farmers with more social networks are more tolerant of others’ untrustworthiness, while farmers with fewer social network resources are less afraid to risk trusting others (Lin, 2001). The second is the “communication” mechanism based on the “contact theory.” Through constant contact, farmers can generalize the cognitive experience gained from interacting objects to others who have no contact but have similar characteristics, thereby changing their attitudes and even giving them trust (Skaalsveen et al., 2020). Based on this, this paper proposes the hypothesis:

H_4_: Social networks play an enhanced moderating role in the process of social trust promoting farmers’ GCT adoption behavior.

Social networks are not only resources, but also the power to produce collective action, which is rooted in the norms of social networks (Ushchev and Zenou, 2020). From the logic of individual action, one is economic rationality and the other is social rationality. The pursuit of economic rationality is to obtain resources through transactions and maximize economic benefits. Social rationality hopes to obtain reputation and social recognition through relationships, and realize value through groups and networks, which relies on the social laws of groups (Lin, 2001). Social networks require people to take collective action values as the rational basis, first give up self-interest, and rely on collective interests to act. Farmers often hope to gain reputation and social recognition through groups and networks. Therefore, the larger the social networks, the greater the social norms that reflect the reputation utility. Based on this, this paper proposes the hypothesis:

H_5_: Social networks play an enhanced moderating role in the process of social norms promoting farmers’ GCT adoption behavior.

**FIGURE 1:**
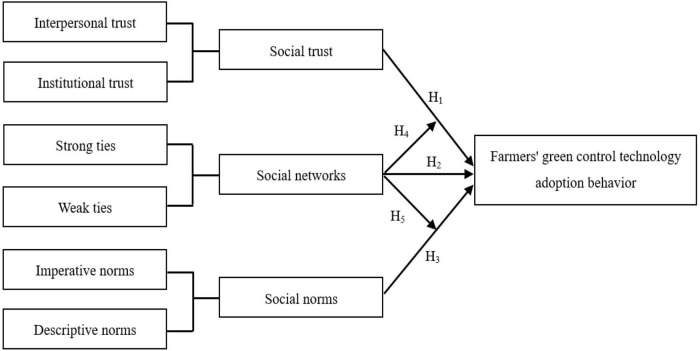
Theoretical analysis framework.

Based on the above theoretical analysis, this paper constructs a theoretical analysis framework, as shown in Figure 1.

## Research design

### Variable assignment

#### Dependent variable

The dependent variable of this paper is farmers’ GCT adoption behavior. GCT includes four technologies: ecological regulation, biological control, physical and chemical control, and scientific drug use. Considering that the current penetration rate of GCT in China is not high, referring to related studies (Gao et al., 2019; Yu et al., 2021), we use the binary variable method to measure farmers’ adoption behavior. When farmers adopt any one or more of these techniques, the value is 1, otherwise the value is 0.

#### Core independent variables

The core independent variables of this paper are three dimensions of social capital, namely, social trust, social networks, and social norms. Referring to relevant literature, this paper measures social trust, social networks, and social norms as follows: ➀ Social trust. The trust degree of farmers to their relatives and friends is used to represent interpersonal trust (Joffre et al., 2020), and the trust degree of village cadres is used to represent institutional trust (He et al., 2018), and the average of them is taken as the final value of this index. ➁ Social networks. The communication frequency with villagers in other villages is used to represent the weak ties, and the communication frequency with villagers in this village is used to represent the strong ties (Kreft et al., 2021), and the average of them is taken as the final value of this index. ➂ Social norms. The number of people in the village where farmers believe that GCT should be adopted is used to represent the imperative norm, and the number of people who adopt GCT in the village where the farmers are located is used to represent the descriptive norm (Li et al., 2022), and the average of them is taken as the final value of this index. All variables are classified according to Likert scale.

#### Control variables

According to the existing research results of influencing factors of farmers’ GCT adoption behavior, gender, age, education level, and health status are selected from the individual characteristics (Khataza et al., 2018; Rezaei et al., 2019; López-Felices et al., 2022), and the number of family labor force, annual family income, planting scale, and distance from village committee (Cheng et al., 2019; Zhou et al., 2019) are selected from the family characteristics, with a total of eight variables as control variables.

#### Instrumental variable

Since social networks are often characterized by “self-selection,” the adoption of GCT and social networks may show a reverse causal relationship, that is, in the process of understanding, learning, adopting, and exchanging GCT, farmers may enhance their social networks observations due to frequent interactions with their relatives and friends (Marvuglia et al., 2022). Therefore, referring to relevant research (Wei et al., 2018), we select “the number of farmers’ New Year’s greetings during the Spring Festival” as an instrumental variable of the social networks to deal with the above-mentioned endogeneity problem. The reasons are as follows: Firstly, New Year’s greetings during the Spring Festival are one of the most important cultural traditions and customs in Chinese society, especially in rural areas. It has a positive impact on the establishment, maintenance and expansion of farmers’ social networks, and has a positive effect on the resources they use from social networks. Secondly, paying New Year’s greetings to relatives and friends during the Spring Festival is mainly restricted by the traditional culture and customs on people’s behavior, but not directly related to farmers’ GCT adoption behavior. Therefore, the number of New Year greetings meets the requirements of tool variables.

The names and definitions of variables are shown in Table 1.

**TABLE 1:** Variables definition and descriptive statistics.

Type	Name	Definition and measure	Mean	Standard deviation
GCT adoption behavior	Whether to adopt	Not adopted = 0; adopted = 1	0.43	0.37
Social trust	Interpersonal trust	Trust in relatives and friends: very distrust = 1; distrust = 2; general = 3; trust = 4; very trust = 5	3.38	0.75
	Institutional trust	Trust in village cadres: very distrust = 1; distrust = 2; general = 3; trust = 4; very trust = 5		
Social networks	Strong ties	Communication frequency with villagers in this village: No contact = 1; less contact = 2; general = 3; more contacts = 4; frequent contacts = 5	3.47	0.82
	Weak ties	Communication frequency with villagers in other village: No contact = 1; less contact = 2; general = 3; more contacts = 4; frequent contacts = 5		
Social norms	Imperative norms	Number of people in the village who think that GCT should be adopted: None = 1; less = 2; general = 3; more = 4; all = 5	3.21	0.98
	Descriptive norms	Number of people adopting GCT in the village: None = 1; less = 2; general = 3; more = 4; all = 5		
Individual characteristics	Gender	Female = 0; male = 1	0.78	0.36
	Age	Respondent’s actual age/years	53.42	12.86
	Education level	Primary school and below = 1; junior high school = 2; senior high school = 3; junior college = 4; university and above = 5	2.46	0.98
	Health status	Very poor = 1, poor = 2, fair = 3, good = 4, very good = 5	4.14	0.90
Family characteristics	Number of labor force	Actual labor force/person	4.33	1.42
	Annual income	Annual income in 2021/million RMB	7.61	4.67
	Planting scale	Planting area/mu	8.45	162.44
	Distance from village committee	Distance from residence to village committee/km	0.96	0.75
Instrumental variable	Social networks Instrumental variable	Number of New Year’s greetings in 2021	5.22	2.34

### Model construction

“Green control technology adoption behavior” is a binary choice problem, so the binary Probit model is constructed, and the expression is as follows:


(1) $$y_{i}=\alpha_{i}+\beta_{1}\,X_{1}+\beta_{2}\,X_{2}+\beta_{3}\,X_{3}+\gamma \,X+\varepsilon_{i}$$

In Eq. 1, _*y_i_*_ is whether farmers have adopted GCT, the value of which has been adopted is “1,” and the value of not adopted is “0.” _*X*_1__ is social trust, _*X*_2__ is social networks, _*X*_3__ is social norms, _*X*_ is control variables. _β_ and _γ_ are the coefficients to be estimated. Among them _β_1__, _β_2__, _β_3__, and _γ_ are used to judge the influence of social trust, social networks, social norms, and control variables on farmers’ GCT adoption behavior. _ε_*i*__ is a random error term.

In order to further verify the moderating effect of social network in farmers’ GCT behavior, this paper uses the interaction term moderating effect analysis model for regression. The specific form of the model is as follows:


(2) $$y_{i}=\alpha_{i}+\beta_{1}^{\prime }\,X_{1}+\beta_{2}^{\prime }\,X_{2}+\beta_{3}^{\prime }\,X_{3}+\beta_{4}\,\left(X_{1}\times X_{2}\right)+\beta_{5}\,\left(X_{1}\times X_{3}\right)+\gamma^{\prime }\,X+\varepsilon_{i}^{\prime }$$

_*X*_1_ × *X*_2__ is the interaction item between social trust and social networks, and _*X*_1_ × *X*_3__ is the interaction item between social norms and social networks. _β_4__ and _β_5__ are used to judge the moderating effect of social networks on social trust and social norms. And the Eq. 2 there may be endogenous problems between social networks and farmers’ GCT adoption behavior. Therefore, the instrumental variable method (IV-Probit) is further used to eliminate the estimation bias caused by endogenous problems.

### Data sources

The data used in this study comes from our team’s questionnaire survey of vegetable farmers in Shandong Province. The reasons for choosing Shandong Province are: Firstly, Shandong Province is the main vegetable producing area in China, and its vegetable planting area and output have ranked first in the country for many years. Secondly, Shandong Province is one of the provinces with frequent pests and diseases, and the situation of vegetable pest control is severe. Thirdly, Shandong Province is a key area for promoting GCT in China, and the number of demonstration counties ranks first in the country (Ministry of Agriculture and Rural Affairs of China, 2021). Fourth, Shandong is the birthplace of Chinese Confucianism, with a strong “relationship culture” and a more obvious role of social capital. Therefore, it is of typical significance to select Shandong Province as the case area.

**FIGURE 2:**
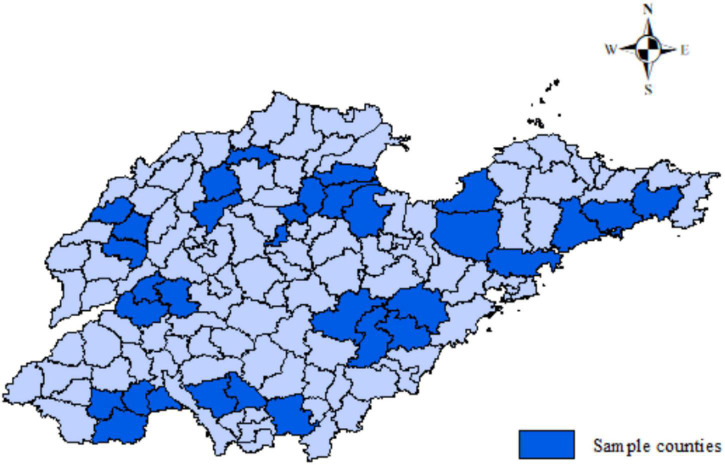
Study area.

The survey was conducted twice, a preliminary survey in November 2021 and a formal survey in February–March 2022. In the first survey, 20 farmers were randomly selected for family interviews in Shandong Province, and the farmers’ adoption of GCT was initially understood, and the questionnaire was revised and improved. In the second survey, stratified sampling and random sampling were adopted. First, two counties were selected in each city, then two towns were randomly selected from each county, and then one or two sample villages were randomly selected in each sample town. Finally, 10 farmers were randomly selected in each village for investigation (Figure 2). Face-to-face interviews were used to gain an in-depth understanding of the survey of individual farmers and their families, social capital and adoption of GCT. A total of 800 questionnaires were distributed in this survey, and 754 valid questionnaires were collected, with an effective rate of 94.3%, excluding invalid questionnaires such as unreturned questionnaires, missed answers or stopped answers in the middle of the survey.

**FIGURE 3:**
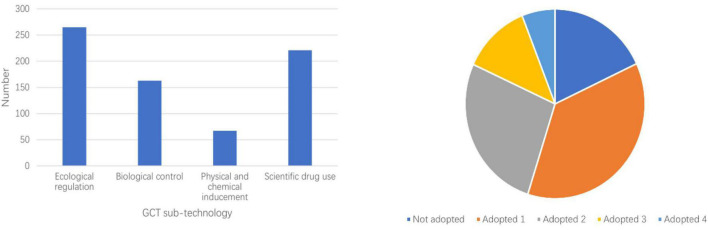
Adoption of farmers’ green control technology (GCT) sub-technology.

According to the situation of farmers’ adoption of GCT (Figure 3), among 754 farmers, 265, 163, 67, and 221, respectively, adopted ecological regulation, biological control, physical, and chemical inducement and scientific drug use, accounting for 35.15, 21.62, 8.89, and 29.31% of the total samples. It shows that farmers adopt different proportions of different sub-technologies in GCT, with the highest proportion of ecological regulation and the lowest proportion of physical and chemical inducement. From the perspective of technology combination, there are 136 farmers who have not adopted any sub-technology, accounting for 18.04%; 278 farmers have adopted 1 sub-technology, accounting for 36.87%; 203 farmers have adopted 2 sub-technologies, accounting for 26.92%; 92 households adopt 3 sub-technologies, accounting for 12.20%; 45 households adopt all 4 sub-technologies, accounting for 5.97%. It shows that the adoption of GCT by farmers is a gradual process, and different farmers have different degrees of adoption of GCT.

Table 2 shows the basic characteristics of the sample farmers. It is not difficult to find that the sample farmers are mainly male, accounting for 78.11%. They are older, with 38.20% of farmers in the 46–55 age group. The education level is relatively low, and 39.39% of the farmers’ education level is junior high school. 67.11% of households have a labor force of 4–6 people. 42.44% of farmers have a planting area of 6–10 mu. The total household income is 51.32% with the highest proportion of farmers with 50,000–100,000.

**TABLE 2:** Basic information of sample farmers.

Variable	Category	Frequency/person	Proportion/%	Variable	Category	Frequency/person	Proportion/%
Gender	Male	589	78.11	Number of labor force	≤3	211	27.98
	Female	165	21.89		4–6	506	67.11
Age	≤45	144	19.10		≥6	37	4.91
	46–55	288	38.20	Annual income//million RMB	≤5	258	34.22
	56–65	181	24.01		5–10	387	51.32
	≥66	141	18.69		≥10	109	14.46
Education level	Primary school	127	16.84	Planting scale/mu	≤5	231	30.64
	Junior high school	297	39.39		6–10	320	42.44
	Senior high school	165	21.88		11–20	134	17.77
	Junior college	111	14.72		21–50	54	7.17
	University and above	54	7.17		≥51	15	1.98

## Estimation results and discussion

### Model inspection

#### Reliability, validity, and correlation test

In this study, the reliability and validity of the data were tested. It can be seen from Table 3 that Cronbach’s α coefficient values of social trust, social networks, and social norms are all greater than the reference standard of 0.7, indicating that the internal consistency of the research data is good. The CR value of each variable is greater than 0.9, which meets the inspection standard. AVE values of all variables are greater than 0.7, which indicates that the aggregation validity of this research scale is good. The square root of each variable AVE is larger than its correlation coefficient with other variables (Table 4), indicating that the research data has good discrimination validity. It can be seen from Table 4 that the correlation coefficients among social trust, social networks, social norms, and GCT adoption behavior are all significantly correlated at the level of 1%.

**TABLE 3:** Reliability and validity test results.

Variable	Cronbach’s α	KMO	CR	AVE
Social trust	0.934	0.892	0.928	0.812
Social networks	0.956	0.904	0.953	0.805
Social norms	0.947	0.878	0.950	0.827

**TABLE 4:** Correlation coefficient matrix.

	1	2	3	4	5	6	7	8	9	10	11	12
1	−											
2	0.022	−										
3	0.005	–0.258	−									
4	–0.013	−0.443*	0.064	−								
5	0.052	–0.073	–0.012	0.069	−							
6	–0.027	0.092	0.358**	0.282	0.201	−						
7	0.049	–0.054	0.170	0.045	0.165	0.177*	−					
8	0.032	0.014	0.221	0.005	0.009	0.022	0.003	−				
9	0.105	−0.089*	0.328**	0.099	0.086	0.072	0.030	0.231*	(0.905)			
10	0.288	−0.050*	0.140	0.212	0.060	0.113	0.042	0.159	0.343**	(0.892)		
11	0.195	−0.105*	0.424**	0.162	0.108	0.101	0.018	0.072	0.321	0.357***	(0.911)	
12	0.174	−0.094*	0.293**	0.183	0.096**	0.166**	0.028	0.102	0.371***	0.362***	0.366***	(0.889)

*, **, and *** indicate significant at the 10, 5, and 1% levels, respectively. The value in brackets is the square root of AVE. (1) Gender; (2) Age; (3) Education level; (4) Health status; (5) Number of labor force; (6) Annual income; (7) Planting scale; (8) Distance from the village committee; (9) Social trust; (10) Social networks; (11) Social norms; (12) GCT adoption behavior.

#### Multiple collinearity test

This paper uses stata 13.0 software for regression analysis. Firstly, considering the multicollinearity problem between variables, the Variance Inflation Factor method (VIF) was used to test the independent variables. The test results show that the VIF values between all independent variables were less than 10, which satisfies the principle of independence, and there is no significant collinearity. Secondly, the social trust, social networks and social norms are separately incorporated into the model, and the model 1–3 is obtained. Finally, the social trust, social networks, and social norms are incorporated into the model, and the model 4 is obtained. From the significance of each model (Table 5), all passed the 1% significance test, indicating that the model has a good degree of fit. Compared with model 1–3, the Pseudo *R*^2^ of model 4 increased to 0.227, which has stronger explanatory power. It shows that model 1–3 does not include the three dimensions of social capital at the same time, resulting in the omission of variables, which will overestimate the influence of the three dimensions of social capital on farmers’ GCT adoption behavior.

**TABLE 5:** Estimated results of the impact of social capital on farmers’ green control technology (GCT) adoption behavior (benchmark regression).

Name	Model 1		Model 2		Model 3		Model 4	
	Marginal effect	Standard error	Marginal effect	Standard error	Marginal effect	Standard error	Marginal effect	Standard error
Social trust	0.047***	0.026	−	−	−	−	0.033**	0.031
Social networks	−	−	0.055***	0.014	−	−	0.038***	0.021
Social norms	−	−	−	−	0.063***	0.037	0.041***	0.026
Gender	0.096	0.026	0.103	0.027	0.111	0.026	0.105	0.025
Age	−0.003**	0.002	−0.001**	0.002	−0.002*	0.002	−0.003*	0.002
Educational level	0.029***	0.058	0.031***	0.060	0.035***	0.065	0.032***	0.062
Health status	0.002	0.005	0.001	0.005	0.003	0.005	0.004	0.005
Number of labor force	0.062***	0.025	0.062***	0.026	0.067***	0.025	0.061***	0.025
Annual income	0.022**	0.004	0.023**	0.005	0.018**	0.005	0.018**	0.004
Planting scale	0.014	0.007	0.016	0.005	0.016	0.006	0.017	0.007
Distance from village committee	–0.002	0.003	–0.002	0.003	–0.002	0.003	–0.002	0.003
Chi-square statistics	173.470***		161.070***		185.215***		197.857***	
Pseudo *R*^2^	0.206		0.193		0.215		0.227	

Probit estimation results report marginal effect, and the standard error is calculated by delta method; *, **, and *** indicate significant at the 10, 5, and 1% levels, respectively.

#### Endogenous test

Considering that there may be endogeneity between farmers’ GCT adoption behavior and social networks, this paper uses IV-Probit model to test endogeneity. Firstly, the endogenous variables are used as the explained variables and the instrumental variables are used as the explanatory variables for regression to obtain the fitting value of the endogenous variables. Then, the fitting value is used as an explanatory variable to introduce the Eq. 1 for regression. In the first stage of model regression, Wald’s endogeneity test results show that the hypothesis that there is no endogeneity is rejected at 1% level. The *F* value of the first stage is 39.47, which exceeds the minimum requirement of *F* = 10 for IV validity, indicating that there is no weak tool variable problem. The IV-Probit two-stage estimation results (Table 6) show that both models 2* and 4* including social networks variables have endogeneity problems (the assumption that all explanatory variables are exogenous cannot be satisfied), and the instrumental variable method is appropriate. Among them, the marginal effect values of the three dimensions of social capital in model 4* are all higher than those in model 4, indicating that if the endogeneity problem is not dealt with, its impact on farmers’ GCT adoption behavior will be underestimated. In addition, because Model 2* only includes social networks variable, its marginal effect value is larger than Model 4*, that is, the impact of social networks on farmers’ GCT adoption behavior is overestimated. Therefore, this paper takes the estimation result of Model 4* as the main explanation result.

**TABLE 6:** Estimated results of the impact of social capital on farmers’ green control technology (GCT) adoption behavior (instrumental variable method).

Name	Model 2*		Model 4*	
	Marginal effect	Standard error	Marginal effect	Standard error
Social trust	−	-	0.036**	0.044
Social networks	0.055***	0.025	0.043**	0.054
Social norms	−	-	0.050**	0.062
Gender	0.427	0.117	0.429	0.124
Age	−0.014**	0.006	−0.015**	0.006
Educational level	0.028***	0.063	0.036***	0.067
Health status	0.007	0.016	0.002	0.018
Number of labor force	0.125***	0.053	0.132***	0.056
Annual income	0.053**	0.014	0.059**	0.011
Planting scale	0.026	0.009	0.038	0.008
Distance from village committee	–0.008	0.010	–0.007	0.011
Wald test value	0.016		0.004	
Prob > χ^2^	0.000		0.000	

Probit estimation results report marginal effect, and the standard error is calculated by delta method; *, **, and *** indicate significant at the 10, 5, and 1% levels, respectively.

### Benchmark regression result analysis

The estimation results of Model 4* show that social trust, social networks and social norms all promote farmers’ GCT adoption behavior, and the significance level is 5%, which verifies the hypotheses H_1_–H_3_. A good level of social trust can make the communication between farmers smoother, reduce the information asymmetry in farmers’ adoption of GCT, improve farmers’ enthusiasm for cooperation with others and their trust in policy implementation, thus encouraging farmers to adopt GCT. Social networks can broaden the channels for farmers to obtain GCT information, increase the possibility of mutual learning, reduce the cost of farmers’ technical information search and technical learning, and promote them to adopt GCT. Social norms often reflect the opinions of most farmers in the village. When most farmers in the village adopt GCT, they will consciously adopt GCT under the dual influence of herd psychology and curiosity psychology.

From the marginal effect results, the probability of farmers adopting GCT will increase by 5.0% for each additional unit of social norms, 4.3% for each additional unit of social networks, and 3.6% for each additional unit of social trust. It shows that social norms play the strongest role, followed by social networks, while social trust plays the weakest role. The possible reason is that social norms are the deepest social embedment, deeply rooted in individual consciousness, guiding farmers’ behavior imperceptibly, and having a deeper and wider influence on farmers’ behavior. Social trust and social networks can only enhance farmers’ understanding of GCT to a certain extent, but they do not play a leading role in the adoption of GCT, so their influence is relatively small.

### Influence of control variables

Model 4* shows that age passed the 5% significant level test. The older the farmers are, the lower their ability to understand and accept new technology, and their motivation and passion for learning are also lower than those of young farmers. Therefore, the lower the possibility of adopting GCT. Education passed the 1% significant level test. Farmers with a higher education level have a certain knowledge reserve, so it is easier to understand the mechanism of GCT, and it is easier to solve problems arising from the implementation of GCT, and the higher the probability of adopting GCT. The number of household labor force passed the 1% significant level test. The implementation of GCT requires household to invest a certain amount of labor force. The greater the number of household labor force, the more energy and ability to learn and implement GCT. The annual household income passed the 5% significant level test. Farmers with higher annual income are more resistant to business risks and have more confidence in adopting GCT.

### Moderating effect analysis

The interaction terms of social networks and social trust, social networks, and social norms are introduced into Eq. 2, respectively, for IV-Probit regression, and model 5 and model 6 are obtained (Table 7). Model 5 shows that the interaction between social networks and social trust has a positive impact on farmers’ GCT adoption behavior at a significant level of 5%, indicating that social networks play an enhanced moderating role in the impact of social trust on farmers’ GCT adoption behavior, and research hypothesis 4 has been verified. The possible explanation is that the distribution of social networks in rural China presents a disparate pattern, and there are frequent interactions and exchanges between relatives and neighbors. Such interactions and exchanges have created a good environment of social trust and enhance the impact of social trust on farmers’ GCT adoption behavior. Model 6 shows that the interaction between social networks and social norms has a positive impact on farmers’ GCT adoption behavior at a significant level of 5%, indicating that social networks play an enhanced moderating role in the impact of social norms on farmers’ GCT adoption behavior, and research hypothesis 5 has been verified. The possible explanation is that the influence of social norms needs to be based on social networks. The more developed farmers’ social networks are, the stronger the role of social norms rooted in social networks will be, so as to better play the role of social norms in farmers’ GCT adoption behavior.

**TABLE 7:** Estimated results of the impact of social capital interaction terms on farmers’ green control technology (GCT) adoption behavior (instrumental variable method).

Name	Model 5		Model 6	
	Marginal effect	Standard error	Marginal effect	Standard error
Social networks × social trust	0.021**	0.008	−	−
Social networks × social norms	−	−	0.029**	0.015
Social trust	0.059**	0.037	0.045**	0.033
Social networks	0.071*	0.025	0.057**	0.040
Social norms	0.080*	0.028	0.063*	0.036
Control variable	Controlled		Controlled	
Wald test value	0.025		0.011	
Prob > χ^2^	0.000		0.000	

Probit estimation results report marginal effect, and the standard error is calculated by delta method; * and ** indicate significant at the 10 and 5% levels, respectively.

### Robustness test

Considering that the selected model will have an impact on the regression results, this paper further selects the Logit grouping regression model to explore the impact of social capital on farmers’ GCT adoption behavior for robustness testing (Table 8). Specifically, according to the average value of farmers’ social networks of 3.47, the farmers’ social networks score greater than or equal to 3.47 are defined as high social networks farmers, and the farmers’ social networks score less than 3.47 are defined as low social networks farmers. Compare the influence of different groups’ social trust and social norms on farmers’ GCT adoption behavior.

**TABLE 8:** Estimated results of the impact of social capital on farmers’ green control technology (GCT) adoption behavior (group regression model).

Name	Model 7		Model 8	
	Low social networks farmers		High social networks farmers	
	Marginal effect	Standard error	Marginal effect	Standard error
Social trust	0.039***	0.012	0.068***	0.021
Social norms	0.061***	0.018	0.075***	0.024
Control variable	Controlled		Controlled	
Pseudo *R*^2^	0.196		0.214	
Chi-square test	75.336***		101.482***	

Probit estimation results report marginal effect, and the standard error is calculated by delta method; *** indicate significant at the 1% levels, respectively.

Table 8 shows that the impact of social trust on the adoption of GCT by two groups of farmers is significant at the 1% statistical level, and the marginal effect is positive. However, from the perspective of influence intensity, the positive impact of social trust on farmers’ GCT adoption behavior of high social networks group is stronger than that of low social networks group farmers, that is, social networks can indeed enhance the positive impact of social trust on farmers’ GCT adoption behavior. The impact of social norms on the adoption of GCT by two groups of farmers is significant at the 1% statistical level, and the marginal effect is positive. However, from the perspective of influence intensity, the positive impact of social norms on farmers’ GCT adoption behavior of high social networks group is stronger than that of low social networks group farmers, that is, social networks can indeed enhance the positive impact of social norms on farmers’ GCT adoption behavior. The estimation results in Table 8 are basically similar to the above analysis results, indicating that the estimation results in this paper are relatively robust.

## Conclusion and policy suggestions

### Conclusion

Based on the survey data of 754 farmers in Shandong Province, this paper empirically analyzes the influence of social capital on farmers’ GCT adoption behavior. The results show that: firstly, the three dimensions of social capital (social trust, social networks, and social norms) all play a role in promoting farmers’ GCT adoption behavior. Among them, social norms play the strongest role, followed by social networks, and social trust is the weakest. Secondly, social networks play an enhanced moderating role in the process of social trust and social norms promoting farmers’ GCT adoption behavior. Thirdly, education level, household labor force, and annual income have significant positive effects on farmers’ GCT adoption behavior, while age has significant negative effects.

### Policy suggestions

Based on the study, this paper puts forward the following suggestions:

1. The government should vigorously cultivate rural non-governmental organizations such as agricultural cooperatives and agricultural associations, and build a good platform for farmers to exchange and learn from each other; use modern information technology to optimize and expand farmers’ access to information, and guide farmers to actively participate in exchanges and experience sharing; encourage farmers to make good use of their own social network resources such as neighbors from the same natural village, friends from different natural villages, relevant intermediaries or service organizations, so as to expand their own social networks.
2. The government should make full use of rural radio, television, and Internet to create a social atmosphere of mutual trust and mutual benefit; actively organize rural cultural activities and production mutual assistance activities, and improve the level of trust among farmers and between farmers and village cadres by showing the working ability and people-friendly style of village cadres in the activities and making use of cooperation and exchange.
3. Encourage large farmers and family farms in the village to adopt GCT, highlight the exemplary role of typical characters, and give full play to its demonstration effect; actively guide the villages to form social norms such as village rules, customs, and habits that are coordinated with them, and strengthen farmers’ reputation utility and social responsibility awareness, so as to give full play to the internal driving force of reputation mechanism in the process of promoting the adoption of green control technologies, and finally guide them to form a good ecological consciousness.
4. The grass-roots governments, village collectives and villagers’ groups should guide farmers to adopt GCT in an orderly manner in batches, starting from young farmers with high education level, large number of family labor force and high annual family income, so as to give play to the leading role of these farmers.

### Limitations and future research

The limitation of this study is that the study area is relatively narrow, and the phenology of the crop production and agronomic behavior of the farmers are expected to be similar. China’s rural areas are vast and have significant differences, so we can consider expanding the scope of research in the future. At the same time, the research data of this study is limited to 2022, and we can try to establish long-term tracking panel data in the future.

## Data availability statement

The original contributions presented in this study are included in the article/supplementary material, further inquiries can be directed to the corresponding authors.

## Author contributions

ZR: writing—original draft. ZF and KZ: reviewing and editing. All authors contributed to the article and approved the submitted version.
